# Constant Temperature Electrochemical Biosensor for SNP Detection in Human Genomic DNA Based on DNA Melting Analysis

**DOI:** 10.1021/acssensors.5c01577

**Published:** 2025-07-16

**Authors:** Skomantas Serapinas, Deimantė Stakelytė, Kornelija Tučinskytė, Miglė Tomkuvienė, Marius Dagys, Dalius Ratautas

**Affiliations:** † Institute of Biochemistry, Life Science Center, 54694 Vilnius University, Saulėtekio al. 7, LT-10224 Vilnius, Lithuania; ‡ Institute of Biotechnology, Life Science Center, Vilnius University, Saulėtekio al. 7, LT-10224 Vilnius, Lithuania

**Keywords:** single-nucleotide polymorphism, gold, biosensor, melting, *CYP2C19*

## Abstract

Detection of single-nucleotide polymorphisms (SNPs) is critical in both bioanalytical science and clinical diagnostics. We present an electrochemical biosensor capable of SNP detection in human genomic DNA immediately following a polymerase chain reaction, eliminating the need for temperature gradients. The biosensor employs a “sandwich” hybridization format in which a target DNA strand binds to an electrode-anchored probe and is interrogated by two allele-specific reporters. By analyzing the kinetic differences in melting rates (*k*_d_) between perfect match (PM) and mismatch (MM) duplexes at constant temperature, we achieved statistically significant differentiation of homozygous and heterozygous alleles (>2σ, *n* = 7). As proof of concept, the biosensor was applied to detect the CYP2C19*17 allele in human saliva samples (*n* = 6), with results confirmed using sequencing. Additionally, melting-rate-derived Gibbs free energy differences allowed for the identification of previously undetected mismatches, suggesting a novel pathway for electrochemical sequencing.

## Graphical abstract

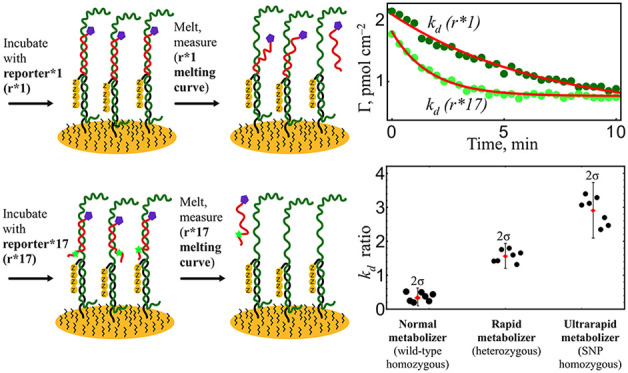

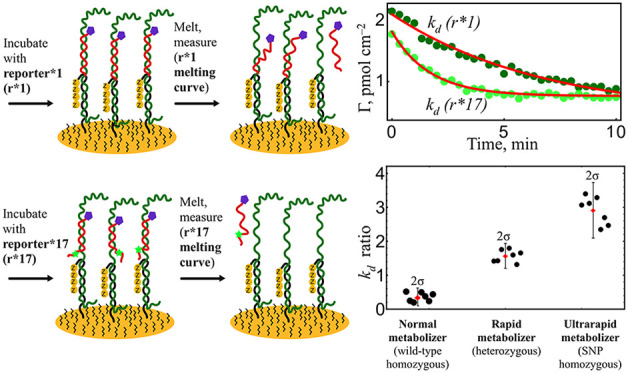

Single-nucleotide polymorphisms (SNPs) are the most common form of genetic variation in the human genome, typically occurring once every ∼300 base pairs and having a minor allele frequency greater than 1%.^1^ Over 84 million SNPs have been identified to date,^2^ and while most are harmless, certain variants are linked to diseases,^3^ aging^4^ and drug metabolism.^5,6^

One clinically significant example is the *CYP2C19* gene, located on chromosome 10, which encodes the cytochrome P450 2C19 enzyme responsible for metabolizing a wide range of xenobiotics. This enzyme is predominantly expressed in the liver, stomach, small intestine, and duodenum.^7^ Clinically relevant variants of *CYP2C19* include loss-of-function (*2–*8), wild-type (WT) (*1), and gain-of-function (*17) alleles. The *17 allele, with a global frequency ranging between 3 and 21%,^8^ affects the metabolism of drugs such as clopidogrel,^9,10^ esomeprazole,^11,12^ and voriconazole,^13,14^ potentially leading to reduced drug efficiencies, treatment unpredictability, and increased toxicity.^15^ Individuals with a heterozygous (*1/*17) or homozygous (*17/*17) genotype are considered rapid or ultrarapid metabolizers, respectively. While research have varied in their stance on *CYP2C19* genotyping,^16^ recent 2024 American Heart Association guidelines recommendations endorse genetic testing for patients undergoing percutaneous coronary intervention or with coronary artery disease.^17^

Electrochemical biosensors are attractive devices for point-of-care determination of analytes of interest. Traditionally, electrochemical biosensors were created for the detection of metabolites such as glucose,^18^ alcohols,^19^ and l-amino acids^20^ usually involving enzymatic catalysts for recognition. Later, biosensors for DNA and RNA detection were also designed and were typically based on DNA or RNA hybridization. For example, Sebuyoya et al. demonstrated an electrochemical biosensor that takes advantage of isothermal LAMP amplification and room temperature hybridization (∼200 bp fragments) for HPV16/18 detection in cervical samples.^21^ Although methods such as TaqMan assays, ddPCR, and SNP microarrays are cost-effective for SNP detection, there remains a strong demand for rapid, low-cost, point-of-care technologies.

SNP detection poses a particular challenge due to the minimal difference between wild-type and variant sequences.^22^ Nevertheless, electrochemical biosensors have been developed for SNP detection. Xu et al. reported an electrochemical biosensor for the detection of epidermal growth factor receptor exon 19 SNP.^23^ They tackled the problem of amplicon (dsDNA) renaturation with phosphorylated primers and lambda exonuclease digestion. Ortiz et al. demonstrated a biosensor for the detection of SNPs associated with rifampicin resistance in *Mycobacterium tuberculosis* using solid-phase primer elongation with ferrocene-labeled nucleotides.^24^ This measurement is “monoploid”, where an electrode array simultaneously measures all 4 possible variants of the point-mutation and compares them. The authors develop their own ferrocenylated master mix, and the discrimination between genes is performed by Klenow polymerase, isothermally on solid phase. Liu et al. showed an SNP analysis platform using multiplexed electrochemical biosensors, highlighting the need to detect several SNPs at once (e.g., *CYP2C19* C680T and G681A).^25^ Han et al. demonstrated a biosensor applicable also to SNP detection. Upon target recognition, dual-DNA–functionalized AuNPs hybridized and bound to SWCNT-DNA via linker strands, forming 3D nanoclusters with apparent electrochemical signals.^26^ Xiao et al. reported a system where an electrochemical sensor was designed for SNP detection based on a triple-stem hairpin DNA probe.^27^ Among these approaches, melting curve analysis remains one of the most widely adopted approaches for distinguishing SNPs. Several notable electrochemical sensors for SNP detection were designed by using this approach. Yang et al. developed a sensor for SNPs in the apolipoprotein E gene, related to Alzheimer’s disease.^28^ The sensor detected SNPs by measuring differences in redox current changes caused by gradually increasing the temperature of WT- and SNP-containing target sequences hybridized to a redox-labeled probe. In this case, a 61-bp target sequence quenches the probe-connected methylene blue (MB) signal. To circumvent the thermal liability of the Au–S bond, the authors employ a trithiolated DNA probe. Chahin et al. showed a similar biosensing platform.^29^ Biosensor utilized ferrocene (Fc)-labeled primer to produce Fc-labeled PCR amplicon. After hybridization with the probe on the electrode surface, the electrode array was subjected to a gradual temperature increase during which the Fc electrochemical signal was measured. The same group also recently demonstrated a biosensing platform, focusing on automation capable to detect several SNPs from human blood.^30^

In this study, we present an improved electrochemical biosensor that eliminates the need for temperature gradients, a major limitation in previous systems. Instead, we optimized the electrode and assay conditions to operate at a constant temperature, enabling SNP detection directly after PCR without additional processing. The biosensor discriminates between wild-type and variant sequences based on differences in melting kinetics. Furthermore, we introduced ZNA-modified probes to enhance electrochemical signal quality. Finally, we show that kinetic melting profiles and derived Gibbs free energy values correlate with the theoretical predictions based on mismatch (MM) position, indicating a potential for broader applications, including the identification of unexpected SNPs within target sequences.

## Results and Discussion

### Principle of Operation of the SNP Sensor

For SNP sensing, we used planar gold electrodes with their surface modified using a ZNA-modified probe (pZNA, nt 21) chemisorbed to the gold surface via thiol chemistry, together with mercaptohexanol for surface blocking. The first iteration sensors were designed using standard DNA probes and performed well with shorter synthetic DNA targets (0–1 nt 5′ overhangs). However, signal transduction was significantly deteriorated when using longer PCR-amplified targets with 14–15 nt 5′ and 4 nt 3′ overhangs resulting from primer design. To overcome this, we have replaced the DNA probe with a 4-spermine unit-containing zip nucleic acid (ZNA-4) probe (formal charge is −10) (see Supporting Information (SI), Probe selection, Figure S1). The ZNA modification was expected to neutralize excess negative charge on the electrode surface, facilitating access of the negatively charged Atto-MB-labeled reporter strand. Previous studies have reported that such charge neutralization reduces ZNA oligonucleotide solubility^31^ and increases duplex melting temperatures.^32^ In terms of surface-bound DNA this effect is referred to as cation-induced nucleic acid collapse and has been demonstrated via addition of Mg­(II),^33^ organic solvents (ethanol)^34^ and freely diffusing polycations.^35^ In our case, probe-bound polycations offer precise, partial charge neutralization localized on the probe molecule.

The assay was designed in the sandwich configuration for compatibility with a large variety of targets. The sensing principle is based on a key property of DNA duplexes: different melting kinetics of perfect match (PM) (r*1-t*1 or r*17-t*17) vs mismatch (r*17-t*1 or r*1-t*17) (Table 3). The melting kinetics of the target-reporter duplex can be observed by using a labeled reporter. In this case, the reporter redox signal is observed via an Atto-MB label (Figure 1). As the target remains captured by the electrode-bound probe, it can be interrogated sequentially with two allele-specific reporters. At least one of the target-reporter duplexes has a mismatch, and it is expected that melting kinetics will differ significantly; the process will be faster. The workflow takes advantage of *ex situ* hybridization (see SI, Figure S2), where the measurement is performed in a separate vessel, while the sample is preserved uncompromised and undiluted by the measurement buffer (Figure 1, Step 0). The sandwich-hybridization stage can be done in two steps to preserve the sample; however, for more rapid one-step hybridization, an excess of reporter was introduced into the sample solution. This approach allows for measurement with a tailored melting solution composition, which may be unsuitable for the hybridization reaction.

**Figure 1:**
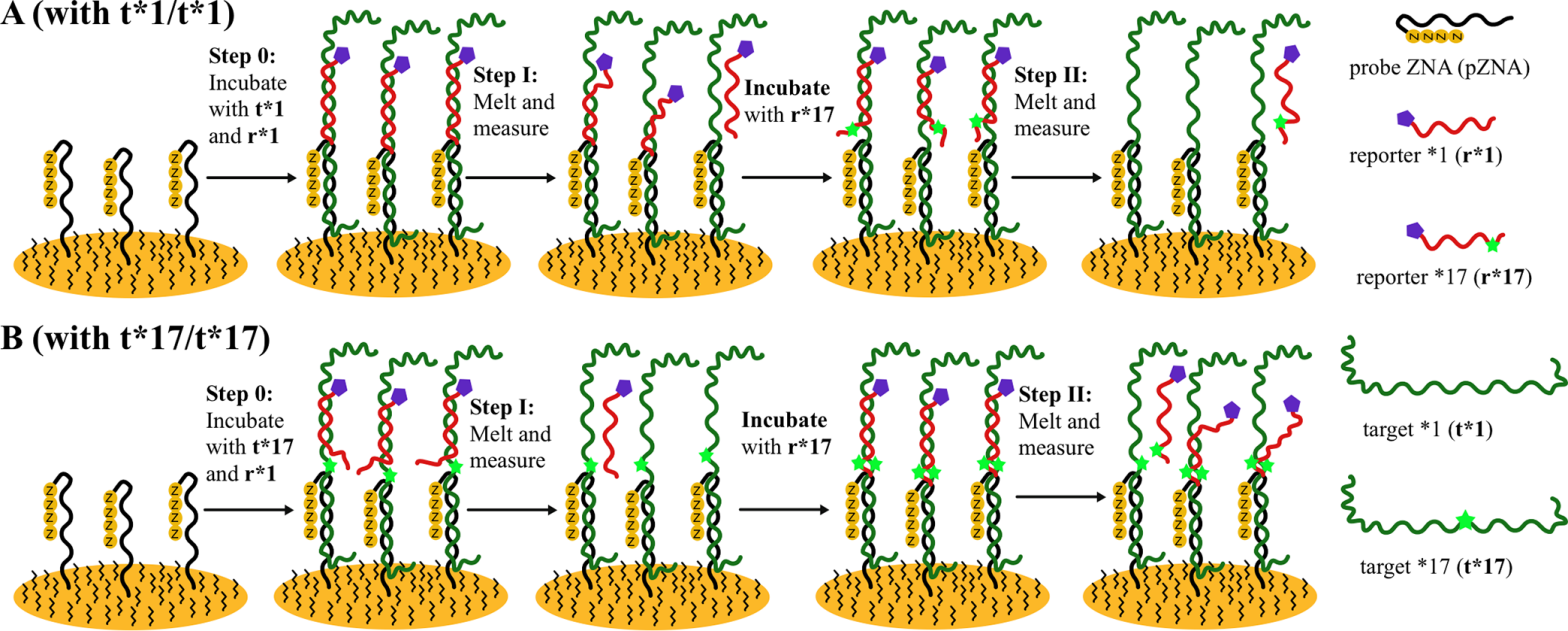
Principle of SNP sensor operation. (A) For the wild-type target (t*1/t*1). At Step 0, the ZNA probe-modified gold electrode is incubated with t*1 and r*1. At Step I, the electrode is placed at an increased fixed temperature, inducing the melting of the r*1–t*1 duplex. Melting kinetics are registered using linear sweep voltammetry (LSV), observing a decrease in the r*1 methylene blue redox signal. Afterward, the r*1-free electrode is incubated with r*17 and at Step II again placed at an increased fixed temperature to observe and record the melting kinetic curve. (B) For SNP target (t*17/t*17). The procedure is the same as in panel (A). After step I and step II, the melting-rate constants *k*_d_ (r*1) and *k*_d_ (r*17) and their ratios *k*_d_ (r*1)/ *k*_d_ (r*17) are determined.

To ensure proper assay performance, NaCl concentration and temperature were optimized (SI, Optimization, Figures S3 and S4). Gold–thiol bonds are temperature-sensitive, so the operating temperature was limited to 42 °C to avoid destabilizing the surface.^36^ Also, from previous work, we were aware that maintaining a higher temperature (i.e., 45 °C) over a reasonable amount of time could cause electrode instability.^37^ High NaCl (>500 mM) slows melting but stabilizes duplex formation and provides better electrostatic screening at the electrode,^38^ while low NaCl (<100 mM) causes a rapid dissociation even of perfect-match duplexes, which compromises detection (SI, Figure S3). Moreover, low NaCl could increase the impact of electrode potential on duplex melting, which is undesirable in our case.^39,40^ By screening a narrow NaCl concentration range (SI, Figure S4), a balance was found and 280 mM NaCl was selected as the most suitable concentration. Additionally, formamide was used as the denaturing agent due to its compatibility with electrochemical detection and its ability to speed up the assay while permitting the use of higher NaCl concentrations.

### Electrochemical Differentiation between Three Possible Diplotypes

The differentiation between the three possible diplotypes, normal metabolizer (NM, t*1/t*1), rapid metabolizer (RM, t*1/t*17), and ultrarapid metabolizer (UM, t*17/t*17)—is essential for genotyping individuals carrying two allele variants. At first, synthetic DNA targets were used to simulate each diplotype. Linear sweep voltammetry (LSV) was selected as the detection method due to its short scan duration and minimal impact on electrode integrity compared to cyclic voltammetry. During melting, LSV curves showed a progressive loss of the Atto-MB redox signal, corresponding to the dissociation of the reporter strand (Figure 2A,B). These curves were fitted using a potential–current function for irreversibly adsorbed species (red lines (inset), Figure 2; SI, eq S1), which enabled the calculation of surface coverage (pmol cm^–2^), while an exponential decay function (eq 1) was then used to fit the determined values to calculate dissociation rate constants (*k*_d_, min^–1^).

**Figure 2:**
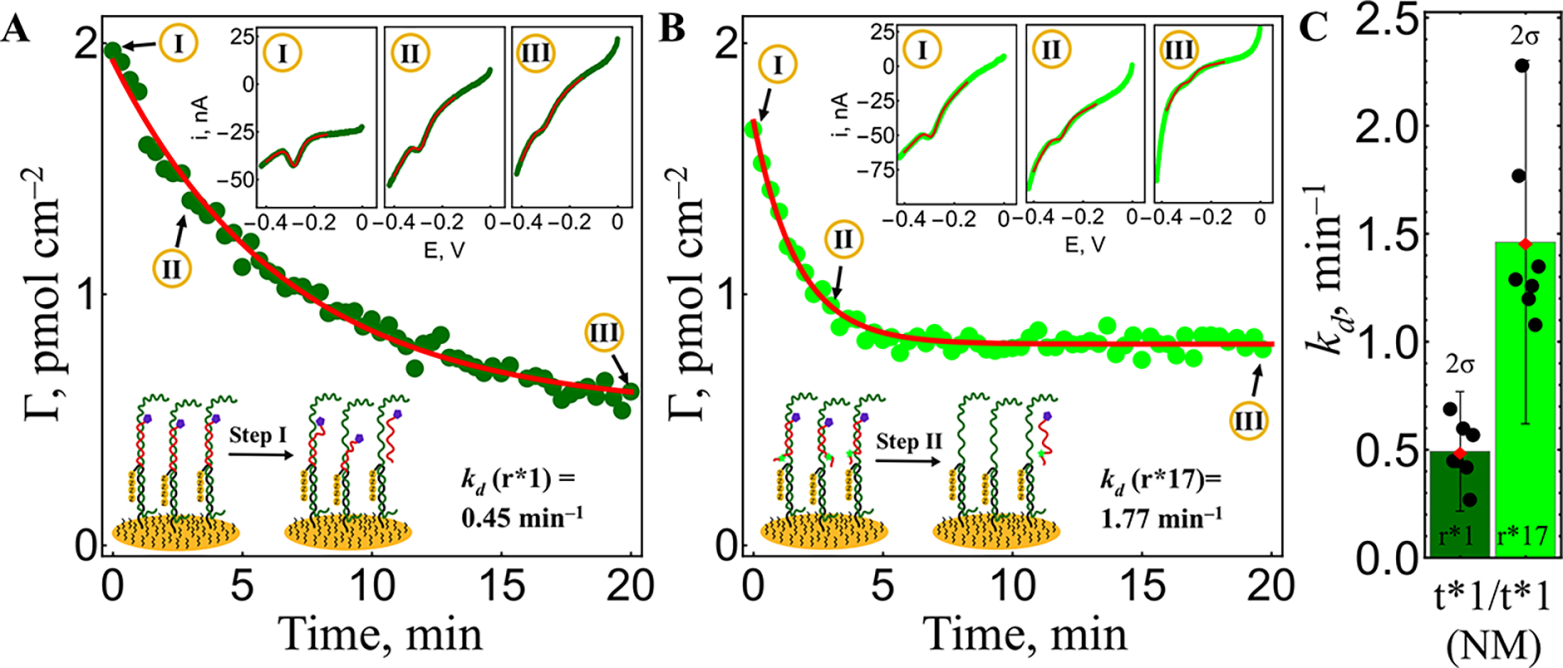
Analysis of the SNP sensor electrode incubated with the NM target. (A) Melting kinetics measured during step I from redox peaks of MB-labeled reporter r*1 using LSV (50 mV s^–1^). Inset: LSV curves at *t* = 0 (I), *t* = 3 min (II), and *t* = 20 min (III). Fitting of the melting kinetics curve to a model to determine the *k*_d_ value for r*1. (B) Melting kinetics were measured during step II from redox peaks of MB-labeled reporter r*17 using LSV. The inset shows LSV curves at *t* = 0 (I), *t* = 3 min (II), and *t* = 20 min (III). Fitting of the melting kinetics curve to a model to determine the *k*_d_ value for r*17. (C) *k*_d_ values for reporters r*1 (dark green) and r*17 (light green) obtained from 7 independent electrodes. Error bars show confidence intervals (±2 standard deviations (SD), 2σ).

Since both reporters, r*1 and r*17, were applied sequentially to the same electrode, their *k*_d_ values are directly comparable. In the example shown (Figure 2), r*1 displays a lower *k*_d_ (higher affinity), while r*17 shows a greater *k*_d_ (lower affinity), consistent with a t*1/t*1 diplotype. This approach was extended to all three diplotypes to calculate the *k*_d_ values for both reporters (Figure 3A). While the *k*_d_ values for the first hybridization (r*1, dark green, Figure 3A) demonstrated an increasing trend (0.48 → 1.2 → 2.4), subsequent hybridization (r*17, light green, Figure 3A) did not provide a clear separation (1.47 → 0.78 → 0.81). To improve the resolution, the ratio of the dissociation rate constants *k*_d_ (r*1)/ *k*_d_ (r*17) was calculated for each electrode (Figure 3B), allowing for robust intraelectrode comparison. The resulting *k*_d_ ratios enabled clear differentiation between diplotypes, with statistically significant separation: 0.35 ± 0.24, 1.56 ± 0.34, and 2.9 ± 0.76 for NM, RM, and UM, respectively (Figure 3B). These ratio intervals were used to precalibrate sensors and define the analysis thresholds (Table 1).

**Figure 3:**
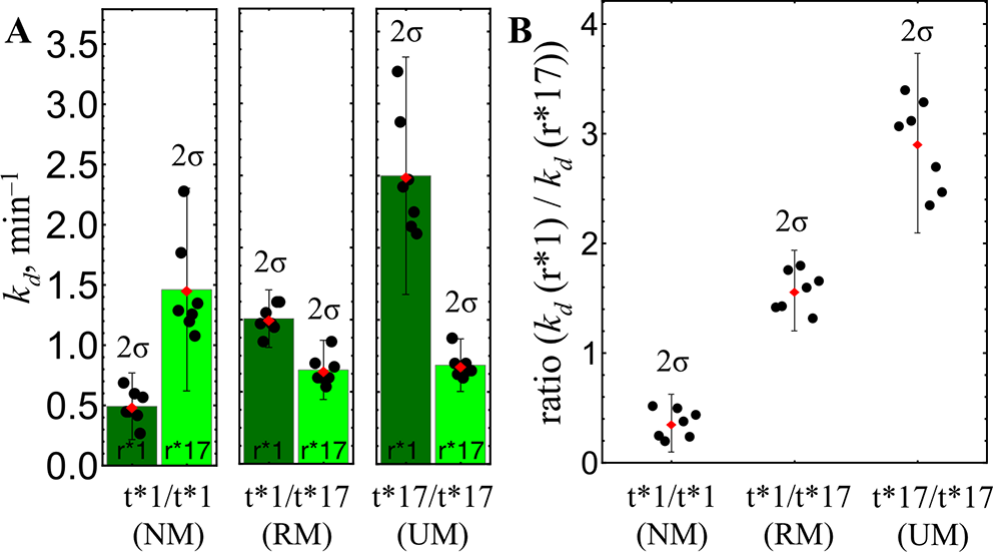
SNP sensor electrode precalibration. (A) *k*_d_ values for reporters r*1 and r*17 for different targets (*p* values between diplotypes: RM|UM pair with r*17 – n. s.; otherwise all *p* < 0.001). (B) Ratio *k*_d_ (r*1)/ *k*_d_ (r*17) for different targets (*p* < 0.0000005). The values were obtained from 7 independent electrodes. Error bars show confidence interval (±2 standard deviations, 2σ).

**Table 1:** Precalibration and Quality Control Data of the SNP Sensor

target	*k*_d_ ratio range (2σ)	Γ_0_ range	*k*_d_ (r*1) range	*k*_d_ (r*17) range
normal metabolizer (t*1/t*1, wild-type homozygous)	0.1–0.62	0.36–3.16 pmol cm^–2^	0.3–3.6 min^–1^	0.6–3.6 min^–1^
rapid metabolizer (t*1/t*17, heterozygous)	1.21–1.93			
ultrarapid metabolizer (t*17/t*17, SNP-type homozygous)	2.09–3.71			

Because the sensors are calibration-free and the *k*_d_ ratio is dimensionless (although no outliers have been observed), it is important to have a sensor quality control (QC) method for future analysis of real clinical samples. We applied a two-tier QC system by combining acceptable surface coverage (Γ_0_) and the *k*_d_ ranges. For Γ_0_, the range was determined using a 3 interquartile range (IQR) fence method,^41^ while the *k*_d_ values were determined during the precalibration (Table 1). In a case where electrode performance values are observed outside of those ranges, the sensor result cannot be trusted, and such measurement should be discarded as a QC-fail.

### Analysis of *CYP2C19* Allele Variants in Human Saliva Genomic DNA

Saliva is an attractive matrix for point-of-care pharmacogenomic testing because it is noninvasive and requires minimal processing. Six anonymized saliva samples (BioIVT) were examined. For each, a 55-bp single-stranded PCR amplicon containing the rs12248560 SNP (CYP2C19*1/*17) locus was generated and applied directly, without purification and any additional pretreatment, to the electrochemical SNP sensor. Of note, asymmetric PCR generating the single-stranded DNA fragment of interest eliminated the need for amplicon denaturation prior to measurements. Parallel sequencing served as the reference. Three samples carried the *1/*1 diplotype (NM), one was *1/*17 (RM), and one was found *17/*17 (UM), covering all possible cases (Table 2). One sample yielded *k*_d_ ratio of 0.79, falling between the NM and RM precalibration ranges (Table 1), and thus was flagged as QC-fail. We suspect that PCR carried contamination, although this was not confirmed. Excluding the QC-fail, sensor genotypes were concordant with sequencing in all samples, demonstrating accuracy and robustness when applied directly to saliva-derived amplicons. Notably, the flagged sample should not be considered a failure *per se*; in real-world settings, variability in sample or electrode preparation is expected, and a well-designed sensor should be capable of detecting such discrepancies via internal QC.

**Table 2:**
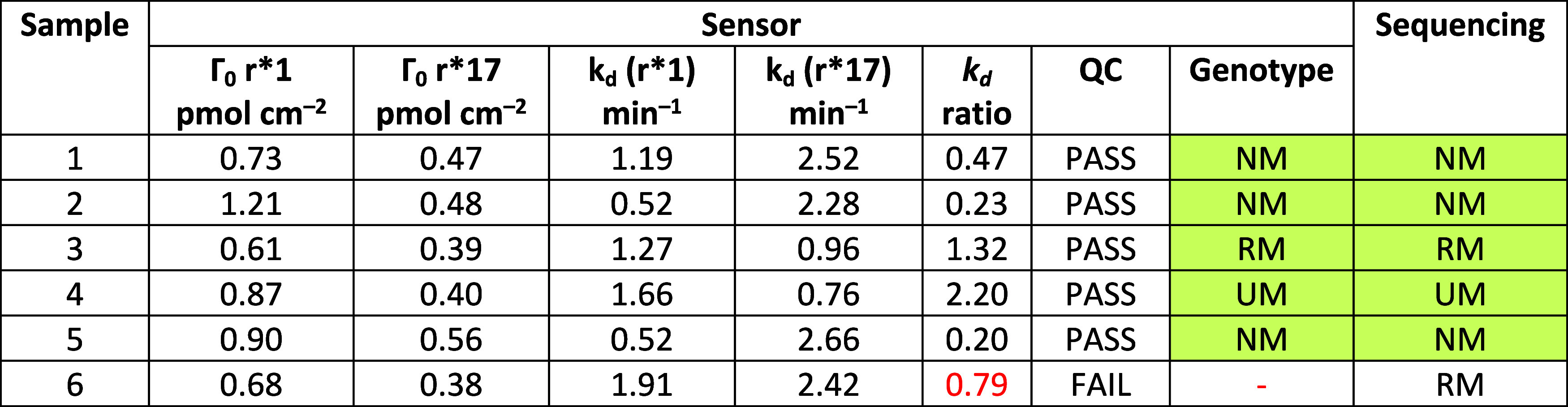
Direct Human Saliva Sample Amplicon Analysis Using an SNP Sensor

### Using Free Energy Differences to Detect Multiple SNPs and Unknown Mutations

While the specific *CYP2C19* sequence used in this study has only one known SNP (1900C > T), many genomic regions contain multiple nearby SNPs, e.g., rs7802307 and rs7802308.^42^ Detecting these with traditional methods can be time-consuming, especially when the variants are unknown or rare. In fluorescence-based DNA microarrays, it is well established that the location of a mismatch has the greatest impact on hybridization signal intensity.^43–46^ Similarly, we found that melting-rate constants (*k*_d_) in our system reflect mismatch affinity and can be used to predict sequence differences.

To extract sequence-specific thermodynamic values, we calculated relative free energy differences (ΔΔ*G*_MM_) by comparing the *k*_d_ values of sample targets to synthetic, perfect-match references (two-target, one-reporter system) (eq 2; SI, Table S3). This isolates the energetic difference introduced by a mismatch. When these experimentally derived ΔΔ*G*_MM_ values are compared to predicted values from thermodynamic models (i.e., DINAmelt), they can be used to estimate the position and potentially the identity of the mismatch. As a proof of concept, we examined a series of synthetic targets: expected SNP (1900C > T) with unexpected mutation (1888C > G) (S1), expected WT only (1900C) (S2), double mismatch (1888C > G; 1900C) (S3), with the perfect match (1900C > T), used as a reference (ΔΔ*G*_MM_ = 0) (S0). In these experiments, we used r*17 as the reporter, forming a perfect match duplex with t*17. The S3 target, containing two mismatches, produced a distinct and significantly elevated ΔΔ*G*_MM_ relative to the single-mismatch targets, demonstrating that multiple deviations from the reference sequence can be determined thermodynamically (Figure 4A). Although the absolute (kcal mol^–1^) values were offset from theoretical predictions by a relative factor of ∼0.55, this discrepancy could be attributed to the heterogeneous nature of the electrode-bound species and the presence of formamide, which is known to alter duplex Gibbs energy by 1.73–5.28 kcal mol^–1^ at 10% on high density microarrays.^46^ Despite this offset, the relative differences between the model targets aligned well with calculated values, enabling discrimination of sequences with single vs double mismatches.

**Figure 4:**
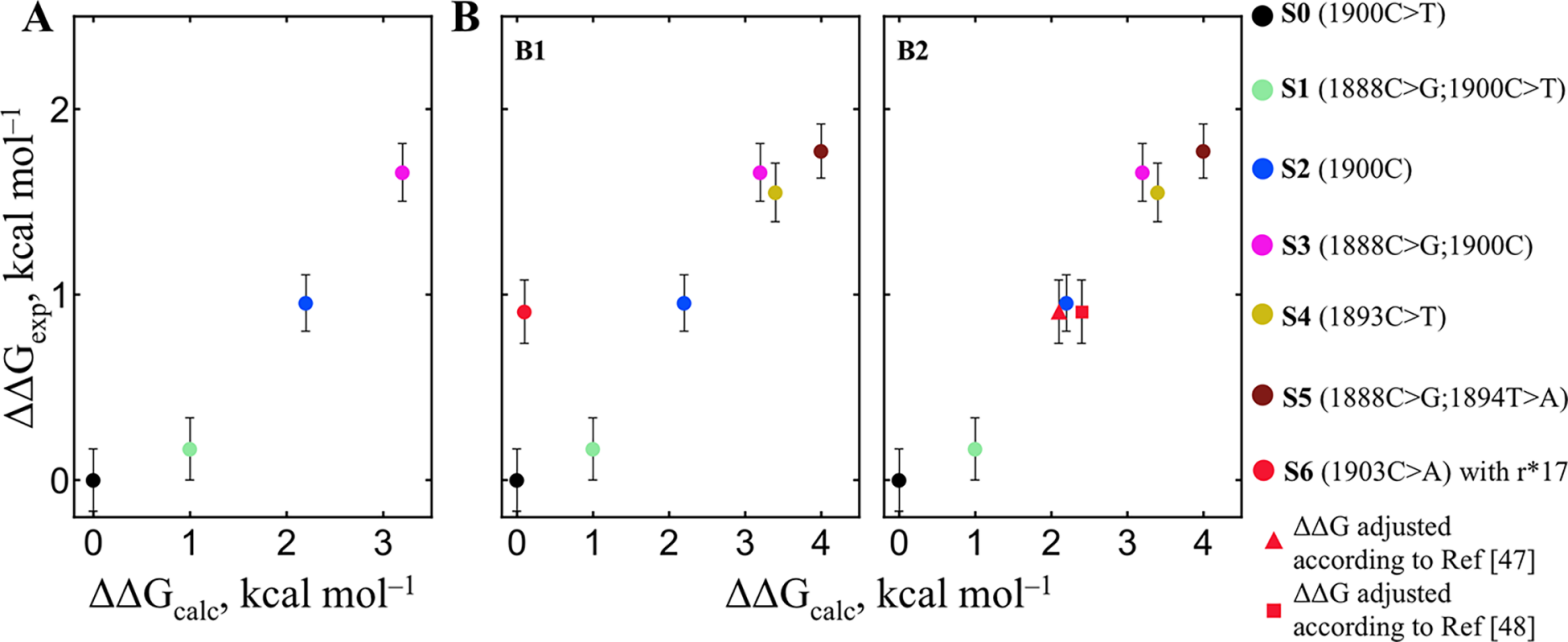
Correlation of measured ΔΔ*G*_MM_ values (ΔΔ*G*_exp_) versus calculated values (ΔΔ*G*_calc_) for the targets having a single mismatch (S1, S2, S4, and S6) and double mismatch (S3, S5). Values are relative to t*17-s-r*17 duplex, which is a perfect match (ΔΔ*G* = 0). (A) ΔΔ*G*_MM_ values for targets tested with/without expected mismatch position 1900C > T. (B) Targets testing with unknown mismatches. Mean ± propagated error; *n* = 3. (Pairs: S0|S1, S2|S6, S3|S4, S3|S5, S4|S5 – n. s., else – *p* < 0.01).

Having obtained these exciting results, we decided to push this approach further and test if it is suitable for the detection of unknown mutations. Additional “unusual” sequences were tested, having a single mismatch: 1893C > T (S4), 1903C > A (S6), and a double mismatch 1888C > G; 1894T > A (S5). The measured ΔΔ*G*_MM_ values were in good agreement with theoretical predictions (Figure 4B1), except for that of S6. We predict that the S6 sequence was behaving unusually. The main difference in S6 was a mismatch at the 3′-terminal position, where the loss of base stacking between the probe and reporter likely caused an underestimation of Gibbs energy. Since DINAmelt does not account for stacking losses (the probe molecule is not included in the calculation at all), the S6 value appeared as an outlier. After applying a correction for *G*|*A* base stacking loss (Δ*G*_stack_ ≈ +2.1 ± 0.3 kcal mol^–1^),^47,48^ the adjusted data point aligned well with model predictions (Figure 4B2).

While the presented approach is promising, several limitations remain. Terminal mismatches, particularly at the 3′ end, can introduce deviations due to base stacking effects, which are not taken into account in standard thermodynamic predictions. Additionally, the presence of formamide in the melting buffer leads to systematic offsets in absolute ΔΔ*G* values, requiring normalization for direct comparison with theoretical models. Variability in the mismatch position relative to the reporter and label may also affect the melting behavior. These limitations can be solved by applying stacking energy corrections, leaving an abasic or similar site in the distal end of the probe or shortening the proximal end of the reporter, and constraining measurements to a single reporter system.

### Future Directions

For clinical applications, we believe two major challenges must be addressed: eliminating the need for thermal cycling (by adopting isothermal amplification methods) and enabling multiplex detection of multiple SNPs simultaneously. Among isothermal approaches, recombinase polymerase amplification has been successfully integrated into a biosensor platform.^30^ For whole-genome amplification, multiple displacement amplification has also proven effective in comparative genomic studies.^49^ Ideally, the hybridization step should be performed at the same assay temperature. In our study, this could not be done due to excessive heating of the electrode. However, replacing standard thiolated probes with trithiolated analogues could improve electrode thermal stability sufficiently to allow sustained exposure at 42 °C.^28^ As for multiplexing, a promising example involves the use of a 16-electrode array to detect multiple SNPs in parallel.^25^ This highlights the potential for electrochemical platforms to eventually achieve high-throughput capabilities comparable to those of spectrophotometric 96-well plate assays for practical clinical use.

## Conclusions

Detection of single-nucleotide polymorphisms was achieved by monitoring the isothermal melting kinetics of DNA duplexes. The biosensor assay was based on a sandwich-hybridization principle: the target DNA was captured by an electrode-bound probe, and the melting-rate constants (*k*_d_) of two allele-specific reporters were measured sequentially. The assay took advantage of the inherent difference in melting rates between perfect match (PM) and mismatch (MM) duplexes, with PM duplexes showing lower *k*_d_, and MM duplexes exhibiting faster dissociation. As a proof of concept, this study focused on a clinically relevant pharmacogenomic variant in the cytochrome P450 family – *CYP2C19*. By employing external sample capture, we enabled the reuse of a single sample amplicon across multiple electrodes, either in parallel or sequentially. The assay is performed entirely at a constant temperature, allowing for simplified instrumentation and uninterrupted melting measurements. Validation was performed using six human saliva genomic samples, successfully distinguishing between CYP2C19 t*1/t*1, t*1/t*17, and t*17/t*17 genotypes. Both allele-specific *k*_d_ values were determined in under 80 min per sample. Unlike traditional SPR-based kinetic systems, our assay focuses solely on dissociation, eliminating the influence of sample concentration and microfluidic variability. Compared to fluorescence-based assays, the required instrumentation is less complex, more cost-effective, and suitable for miniaturization—offering potential for multiplexing via parallel electrodes, particularly for high-value pharmacogenetic targets. Additionally, this study also uncovered a new application of the method: the prediction of one or more nucleotide substitutions based on target affinity. We demonstrated that differences in the Gibbs free energy, derived directly from *k*_d_ values, can be used to detect and characterize additional mismatches. This insight opens a promising path toward electrochemical pseudosequencing, where unknown point mutations could be identified through dissociation-based Gibbs energy profiling.

## Experimental Section

### Instrumentation

SensoQuest Labcycler was used for PCR. A Gamry Reference 600+ potentiostat for electrochemical measurements was used with a Pt wire counter electrode and Ag/AgCl (3 M KCl) reference electrode.

### Reagents, Solutions, Materials, and Electrodes

Felt pads and 0.3 μm alumina suspension were purchased from Buehler. The gold disc (*d* = 2 mm) working electrodes were purchased from BASi. Nuclease-free water, Sodium chloride, tris­(2-carboxyethyl)­phosphine hydrochloride (TCEP), and 6-mercapto-1-hexanol were purchased from Sigma. Disodium hydrogen phosphate was purchased from Alfa Aesar. Formamide, magnesium chloride hexahydrate, and sulfuric acid were purchased from Merck. Tris was obtained from Roth. Tween-20 was purchased from Ferak Berlin. Yeast RNA was purchased from Roche. All reagents were analytical grade. All synthetic oligonucleotides used in this work were purchased from Metabion AG. The sequences are given in Tables 3, S1, and S2. The obtained freeze-dried oligonucleotides were dissolved in 10 mM Tris buffer, pH 8.00. The storage concentration of the target sequences was 400 μM and of the reporter sequences, 100 μM. The ZNA probes were obtained as a 100 μM solution in water, which was aliquoted prior to freezing and used as is. These solutions were kept at −20 °C. Human saliva samples were purchased from BioIVT (Human Head and Neck-Related Other Biofluids).

**Table 3:**
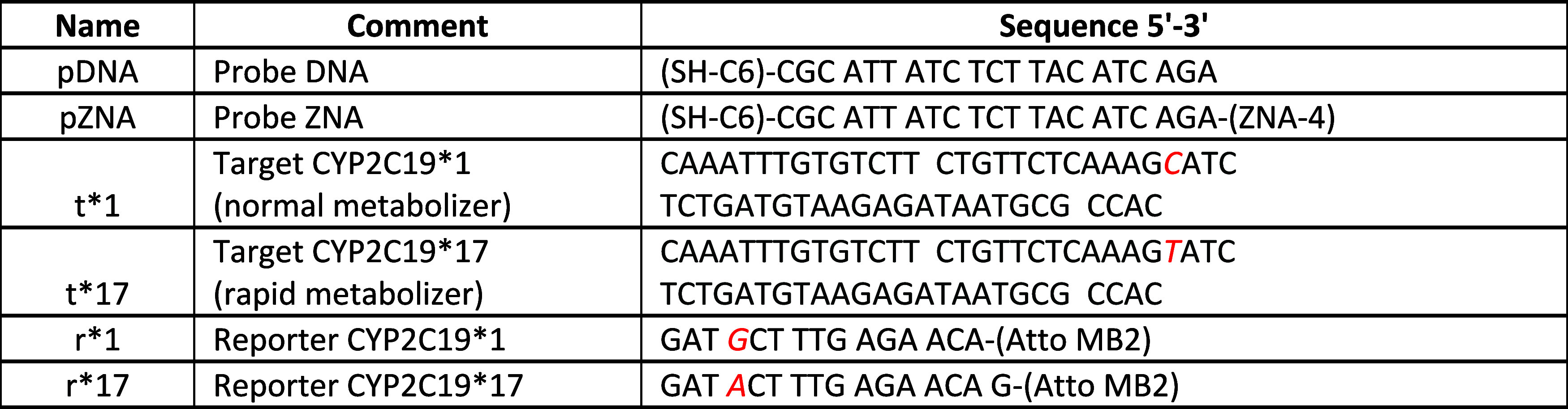
Sequences Used for CYP2C19*1/*17 Genotyping^a^

a Nucleotide substitution position (1900C > T) and related reporter nucleotide position (G > A) are highlighted in red.

### Electrode Preparation

The electrodes were polished on a felt pad wetted with deionized water and a 0.3 μm alumina suspension and sonicated in water for 5 min in an ultrasound bath. The electrochemical cleaning was performed by running 20 CV scans from – 0.2 to 1.75 V at a scan rate of 0.3 V s^–1^ in 500 mM H_2_SO_4_ solution. The electrodes were kept in H_2_SO_4_ and thoroughly rinsed with deionized water prior to modification. The probe oligo (pZNA or pDNA) solution was prepared using TCEP (33.3 μM thiolated oligo, 20 mM TCEP, 10 mM Tris, and 100 mM MgCl_2_, pH 7.2). This mixture was kept at room temperature for 30 min. The probe oligomers were diluted to a final concentration of 0.125 μM (10 mM Tris, 100 mM MgCl_2_, pH 7.2). Gold electrodes were immersed and kept in the probe oligo solution ([pZNA] = 0.125 μM; [pDNA] = 0.25 μM) for 40 min at room temperature. Afterward, the electrodes were submerged in 7.3 mM aqueous 6-mercapto-1-hexanol solution for 30 min for surface blocking, at RT. Subsequently, the electrodes were washed with 20 mM PBS, 3 M NaCl, pH 7 buffer solution, and left overnight in the same solution.

### Hybridization

The electrodes were briefly washed using a solution containing 20 mM PBS and 200 mM NaCl, pH 7. The hybridization of Target and Reporter sequences with the immobilized probe on the gold disc electrode were performed as one step in a single test tube in 5XSSC/0.1% SDS solution containing 100 nM Target ssDNA or at least 0.1 v/v PCR product and 170 nM Reporter ssDNA (the first hybridization performed with r*1 and the second with r*17) sequences and 0.2 mg/mL yeast RNA at 39 °C for at least 20 min. The final volume of the hybridization solution was at least 100 μL. No additional purification or denaturation steps were performed for the PCR product. The electrodes were briefly rinsed in the melting buffer prior to a second measurement with r*17 to ensure only minuscule previous reporter (r*1) presence.

### Melting Measurements

The melting experiments were carried out in a jacketed cell in 20 mM PBS, 280 mM NaCl, 10% Formamide, and 0.05% Tween-20, pH 7 (Melting buffer) at 42 °C, unless stated otherwise. The solution was stirred constantly (200 rpm). LSVs were recorded from 0 to −0.42 V at a scan rate of 0.05 V s^–1^. For every melting measurement, a total of 60 LSVs were recorded at intervals of 20s. The melting curves were fitted according to an exponential dissociation model, ignoring reassociation based on the Polanyi–Wigner equation^50,51^

1 $$\Gamma \left(t\right)=\left(\Gamma_{0}-\Gamma_{\infty }\right)e^{-k_{d}\times t}+\Gamma_{\infty }$$

Here, Γ_0_ – starting surface coverage of reporter (pmol cm^–2^), Γ_∞_ – final surface coverage of reporter which is left hybridized (pmol cm^–2^), *k*_d_ – dissociation rate constant (min^–1^), *t* – time (min).

After the first measurement, the electrode was submerged in the second hybridization solution (as above) and measured again to obtain the dissociation rate constant for the second reporter. The melting buffer (at least 8.0 mL was used for measurements) was not replaced between the electrodes or measurements.

### Measurement of Mismatch Free Energy

Since the hybridization during the experiment is negligible (only melting occurs), then *k*_d_ ∝ e^–*E*_a_/*RT*^. ΔΔ*G*_MM_ can be calculated from existing *k*_d_ (s^–1^) rate constants using the formula^48^

2 $$\Delta \Delta G_{\mathrm{MM}}=\Delta E_{a}=\mathrm{RT}\;\ln\left(k_{d\left(\mathrm{MM}\right)}/k_{d\left(\mathrm{PM}\right)}\right)$$

Here, *R* – gas constant (1.9872 cal·K^–1^ mol^–1^), *T* – temperature (315.15 K).

The ΔΔ*G*_calc_ values were obtained using UnaFold Two State Melting-hybridization,^52^ which could be freely calculated using The UNAFold Web Server. Using *T* = 42 °C, 280 mM Na^+^, and 0.01 mM C_T_ in Oligo Mode. Only the reporter-complementary portion of the target was taken into account for calculations. By subtracting Δ*G*_MM calc_ – Δ*G*_PM calc_ = ΔΔ*G*_MM calc_, the calculated ΔΔ*G*_calc_ values were obtained. The experimental values were obtained from t*17-s and S1–S6 targets.

### Human Saliva Genomic DNA Asymmetric PCR Amplification to Generate Single-Stranded DNA Fragments of Interest

PCR was performed directly on human saliva samples, using DreamTaq DNA Polymerase in its reaction buffer (as recommended by the manufacturer Thermo Scientific), 0.25 μM primers (Table S1), and 0.2 μL of saliva sample to generate a 55 bp PCR product spanning the SNP of interest (rs12248560). PCR was carried out in thin-walled test tubes in a thermocycler under the following conditions: initial denaturation at 95 °C for 2 min, followed by 35 cycles of denaturation at 95 °C for 30 s, annealing at 57.8 °C for 30 s, and extension at 72 °C for 1 s. Further, ssDNA copies of the strand of interest were generated by asymmetric amplification by adding 0.5 μM forward primer and 1.25U DreamTaq polymerase directly into the reaction mixture and applying the second round of thermocycling conditions as described above, up to 35–50 additional cycles. In parallel, 462 bp long dsDNA PCR products,^53^ spanning the site of interest, were produced, followed by nanopore-sequencing by SeqVision.

### Statistics

For reliable diplotype determination, a minimum separation of 2σ was targeted to ensure high-confidence discrimination. To aid data interpretation, additional multiple comparisons were performed using one-way ANOVA followed by Tukey’s HSD test, with p < 0.01 being considered statistically significant. In Figure 3, only target sequences were compared as the reporter sequence is known and does not require measurement. In Figure 4, the mean, propagated standard deviation (SD), and number of measurements and groups were used instead of experimental replicates, as only the mean experimental free energy values (kcal mol^–1^) were available.

## Supplementary Material



## Notes

Tables of all used nucleotide sequences (including those for ΔΔ*G* measurements); *ex situ* hybridization workflow figure; supporting figures for probe selection and optimization; and supporting equation (PDF)
