# Supplementary material for: Constant Temperature Electrochemical Biosensor for SNP Detection in Human Genomic DNA Based on DNA Melting Analysis
Source: ACS Sens. 2025 Jul 16;10(9):6819–27. doi: 10.1021/acssensors.5c01577 (PMC12481571; doi:10.1021/acssensors.5c01577)
Supplement: Supplementary file 1 [file se5c01577_si_001.pdf]

Constant temperature electrochemical biosensor for SNP detection in human genomic DNA based on DNA melting analysis

Skomantas Serapinas,[a]* Deimantė Stakelytė,[a] Kornelija Tučinskytė,[b] Miglė Tomkuvienė,[b] Marius Dagys,[a] and Dalius Ratautas [a]*

[a] Institute of Biochemistry, Life Science Center, Vilnius university, Saulėtekio al. 7 LT-10224, Vilnius, Lithuania
[b] Institute of Biotechnology, Life Science Center, Vilnius university, Saulėtekio al. 7 LT-10224, Vilnius, Lithuania
* Corresponding authors. Skomantas Serapinas (skomantas.serapinas@gmc.vu.lt) and Dalius Ratautas (dalius.ratautas@gmc.vu.lt)

Supporting information

Table S1. All sequences used.

Name	Comment	Sequence 5'-3'
pDNA	Probe DNA	(SH-C6)-CGC ATT ATC TCT TAC ATC AGA
pZDNA	Probe ZNA	(SH-C6)-CGC ATT ATC TCT TAC ATC AGA-(ZNA-4)
t*1-5	Target CYP2C19*1 (containing only 5’ overhang)	CAAATTTGTGTCTT CTGTTCTCAAAGCATC TCTGATGTAAGAGATAATGCG
t*1	Target CYP2C19*1	CAAATTTGTGTCTT CTGTTCTCAAAGCATC TCTGATGTAAGAGATAATGCG CCAC
t*17-5	Target CYP2C19*17 (containing only 5’ overhang)	CAAATTTGTGTCTT CTGTTCTCAAAG*T*ATC TCTGATGTAAGAGATAATGCG
t*17	Target CYP2C19*17	CAAATTTGTGTCTT CTGTTCTCAAAG*T*ATC TCTGATGTAAGAGATAATGCG CCAC
r*1	Reporter CYP2C19*1	GAT *G*CT TTG AGA ACA-(Atto MB2)
r*17	Reporter CYP2C19*17	GAT *A*CT TTG AGA ACA G-(Atto MB2)
Fw	Forward PCR primer	CAAATTTGTGTCTTCTGTTCTC
Rev	Reverse PCR primer	GTGGCGCATTATCTCTTACATC
t*1-s	Flush Target *1 (containing no overhangs)	CTGTTCTCAAAGCATC TCTGATGTAAGAGATAATGCG
t*17-s	Flush Target *17 (containing no overhangs)	CTGTTCTCAAAG*T*ATC TCTGATGTAAGAGATAATGCG
S1	$\Delta\Delta G_{MM}$ testing (1888C>G;1900C>T)	*G*TGTTCTCAAAGTATC TCTGATGTAAGAGATAATGCG
S2	$\Delta\Delta G_{MM}$ testing (1900C)	CTGTTCTCAAAG*C*ATC TCTGATGTAAGAGATAATGCG
S3	$\Delta\Delta G_{MM}$ testing (1888C>G;1900C)	*G*TGTTCTCAAAG*C*ATC TCTGATGTAAGAGATAATGCG
S4	$\Delta\Delta G_{MM}$ testing (1893C>T)	CTGTT*T*TCAAAGTATC TCTGATGTAAGAGATAATGCG
S5	$\Delta\Delta G_{MM}$ testing (1888C>G;1894T>A)	*G*TGTTC*A*CAAAGTATC TCTGATGTAAGAGATAATGCG
S6	$\Delta\Delta G_{MM}$ testing (1903C>A)	CTGTTCTCAAAGTAT*A* TCTGATGTAAGAGATAATGCG

Table S2. Target sequences used for $\Delta\Delta G_{MM}$ measurements.

Name	Sequence 5'-3'	$\Delta\Delta G_{MM}$ (calc), kcal mol^{-1}
t*17-s	C TGT TCT CAA AGT ATC TCT GAT GTA AGA GAT AAT GCG	-
S1	*G* TGT TCT CAA AGT ATC TCT GAT GTA AGA GAT AAT GCG	1.00
S2	C TGT TCT CAA AG*C* ATC TCT GAT GTA AGA GAT AAT GCG	2.20
S3	*G* TGT TCT CAA AG*C* ATC TCT GAT GTA AGA GAT AAT GCG	3.20
S4	C TGT T*T*T CAA AGT ATC TCT GAT GTA AGA GAT AAT GCG	3.40
S5	*G* TGT TC*A* CAA AGT ATC TCT GAT GTA AGA GAT AAT GCG	4.00
S6	C TGT TCT CAA AGT AT*A* TCT GAT GTA AGA GAT AAT GCG	0.10

Probe selection

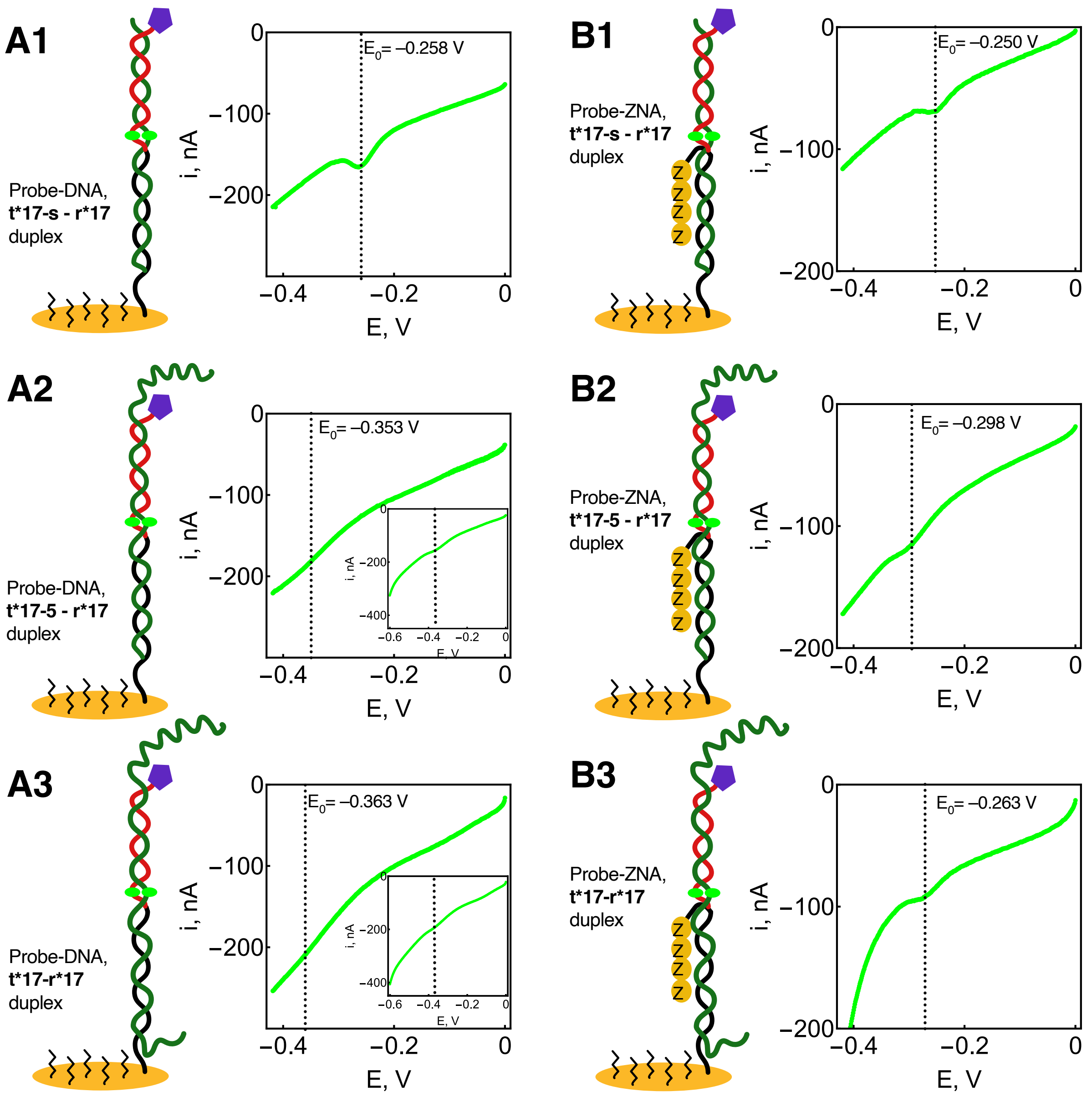

Figure S1. Probe selection experiments for SNP sensor electrode, in all cases the formed hybrid was a perfect match. **A1–A3**. Electrode analysis using LSV when electrode is modified with pDNA. Electrode is hybridized with t*17-s (A1), t*17-5 (A2) and t*17 (A3). **B1-B3**. Electrode analysis using LSV when electrode is modified with pZNA. Electrode is hybridized with t*17-s (A1), t*17-5 (A2) and t*17 (A3). LSV cathodic peak shift could clearly be observed to a more positive values when pZNA is used. Green dots in the figure show the SNP location.

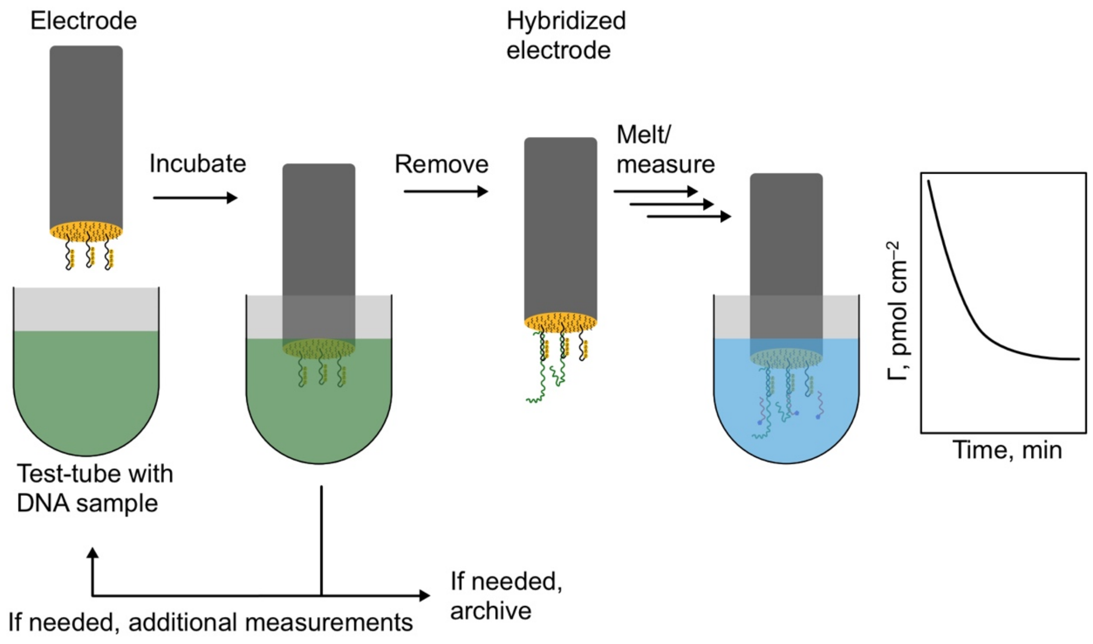

Figure S2. Workflow on the designed sensor including *ex-situ* hybridization. Electrode is incubated in a sample container, while the measurement is performed in a separate vessel. The sample is preserved uncompromised and undiluted by the measurement buffer, thus it is suitable for storage and additional measurements.

Optimization

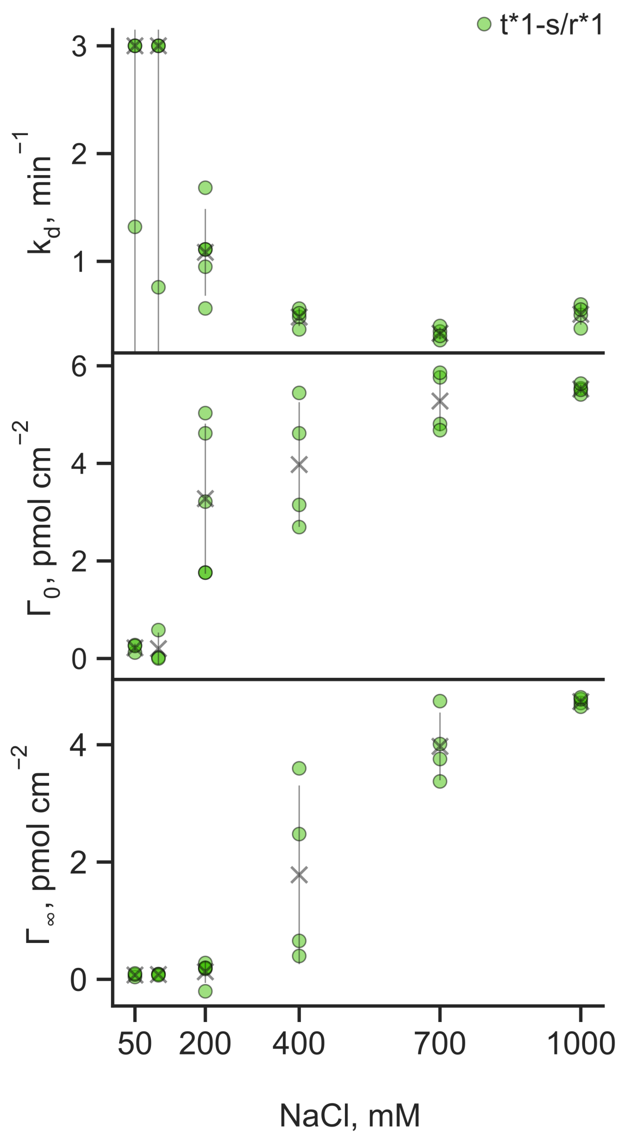

Figure S3. [NaCl] optimization experiments **with the DNA probe** done for model perfect match duplex without overhangs t*1-s–r*1. Results show that the melting was instantaneous (as the hybridization was performed externally) at 50-100 mM NaCl and became too slow to measure at 700–1000 mM. We also see a considerable baseline using 400 mM NaCl at Γ_∞, indicating that melting efficiency is unsatisfactory. Conditions: 42°C, 10% FA, 20mM PB, pH 7.0, CV 0.1V:-0.4V:0.1V, 50mV/s, 1*CV/min, intermittent stirring.

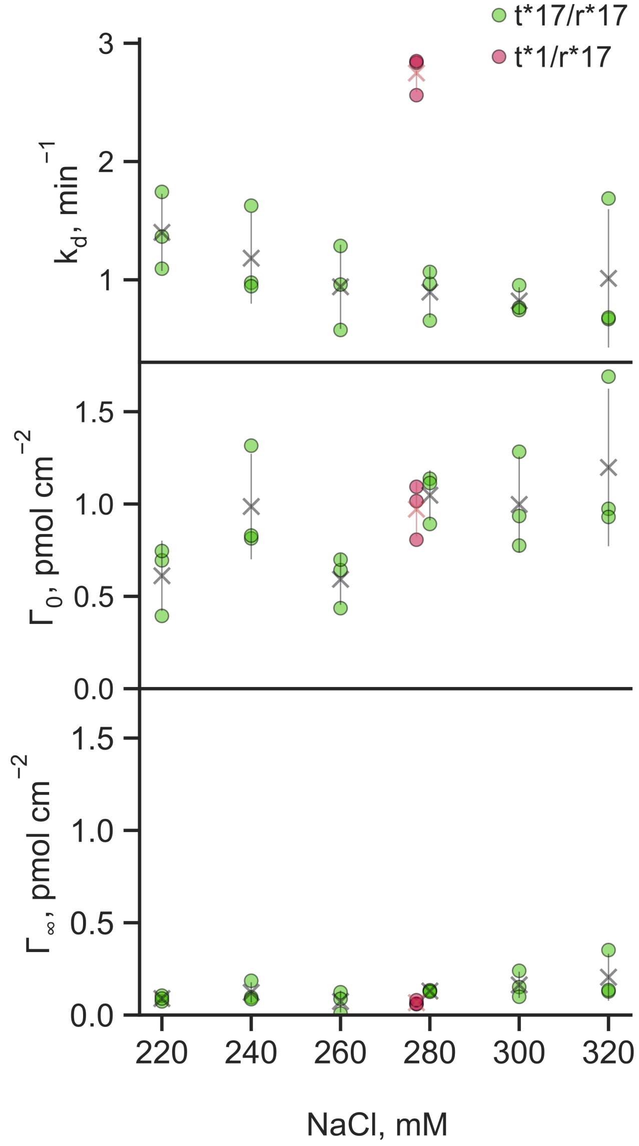

Figure S4. [NaCl] optimization experiments **with the ZNA probe** done for perfect match (t*17–r*17) and mismatch (t*1-r*17) duplexes containing overhangs. The melting conditions were tuned to minimize the k_d of a perfect match pair (t*17-r*17), while ensuring high k_d for mismatch pair (t*1-r*17). The NaCl concentration was screened in a narrow window of 220-320mM and overall good performance was observed in the entire range. 280mM NaCl was selected as the k_d values start to trend up at lower concentrations of NaCl and at higher concentrations of NaCl we will begin to see an increased baseline signal. Conditions: 42°C, 10% FA, 20mM PB, pH7, LSV 0 V:-0.42V, 50mV/s, 3 LSV/min, continuous stirring.

Mismatch prediction

Table S3. Target sequences used for ΔΔG measurements. For simplicity, only the relevant part of the sequence, for which the calculation is performed is shown, leftover part is named here Seq. For complete sequences see Table S2.

Name[1]	$\Delta\Delta G_{MM}$[2]	± ΔΔG[3]	$\Delta\Delta G_{calc.}$[4]	Duplex with r*17; R= 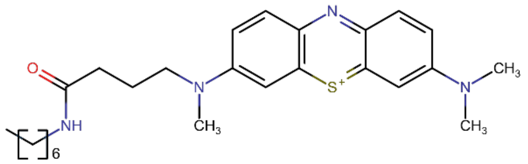
**t*17-s** (1900C>T)	-	-	0	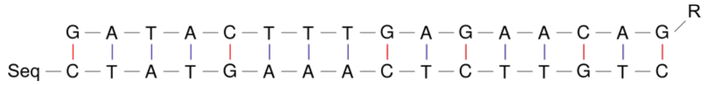
**S1** (1888C>G; 1900C>T)	0.168	0.168	1.000	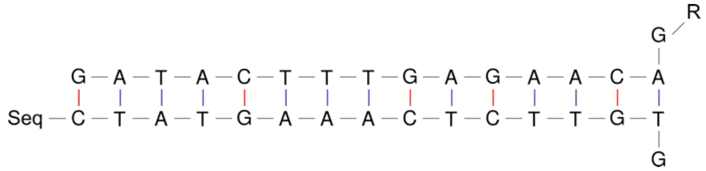
**S2** (1900C)	0.955	0.152	2.200	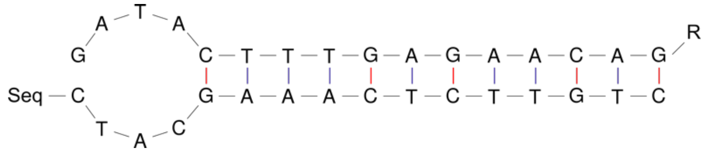
**S3** (1888C>G; 1900C)	1.659	0.156	3.200	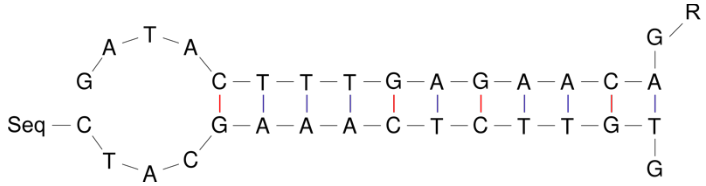
**S4** (1893C>T)	1.551	0.158	3.400	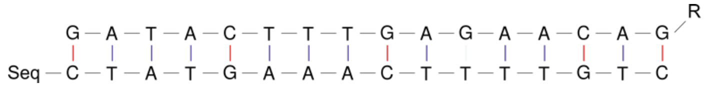
**S5** (1888C>G; 1894T>A)	1.774	0.146	4.000	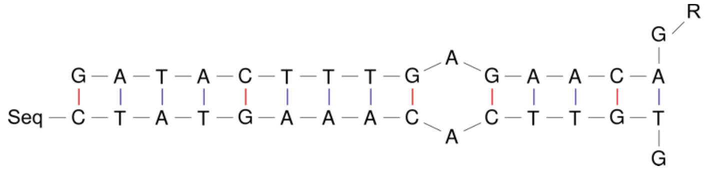
**S6** (1903C>A)	0.908	0.171	0.100	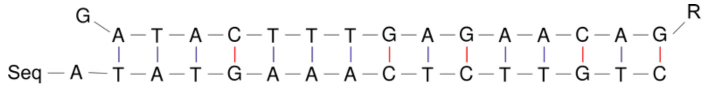

1. Position in accordance with NC_000010.11:g.9476(1900C>T) HGVS Nomenclature 2. Experimental values, kcal mol^{-1}; 3. Propagated error, kcal mol^{-1}; 4. Values calculated from DINAmelt results, kcal mol^{-1}

Data Fitting

Potential-current function of surface-adsorbed redox-active species

$$i(E) = \frac{n^2F^2v\ Olig\ Exp\left[\frac{nF}{RT}(E-E_0)\right]}{RT\left(1+Exp\left[\frac{nF}{RT}(E-E_0)\right]\right)^2} + a_0 + a_1(E-E_0) + a_2(E-E_0)^2 \qquad \text{(Eq.S1)}$$

Here, i – current, n – number of electrons participating in the reaction (2), ν – potential scan rate (V s^{-1}), F – Faraday constant (96485 C mol^{-1}), Olig – MB-labeled oligo amount on the electrode surface (product of AΓ) (mol), R – molar gas constant (8.3145 J K^{-1} mol^{-1}), T – absolute temperature (315.15 K), E – scanned electrode potential (V), E_0 – standard redox potential of MB (V), α_0 – α_2 – background current coefficients. Model derivation is described in Bard[1] and data fitting procedures are described extensively in our previous papers.[2,3] For context, the absolute DNA dissociation rate constant has been reported via surface plasmon resonance to be in the range of 3×10^{-5} – 2×10^{-3} s^{-1} (0.0018 – 0.18 min^{-1}) at 25°C on heterogeneous phase.[4]

References

(1) Faulkner, L. L.; Bard, A. J., 2nd ed.; Wiley: New Yor, 2001, p. 590.
(2) Serapinas, S.; Gineitytė, J.; Butkevičius, M.; Danilevičius, R.; Dagys, M.; Ratautas, D. *Biosens. Bioelectron.* **2022**, *213*, 114475. https://doi.org/10.1016/j.bios.2022.114475.
(3) Gineitytė, J.; Serapinas, S.; Ratautas, D. *Electrochim. Acta* **2024**, *507*, 145146. https://doi.org/10.1016/j.electacta.2024.145146.
(4) Simon, L.; Gyurcsányi, E. R. *Electrochim. Anal. Chim. Acta* **2019**, *1047*, 131–138. https://doi.org/10.1016/j.aca.2018.09.048
